# Dr. Charles H. Parry, in Reply to Dr. Wilson Philip

**Published:** 1821-07

**Authors:** 


### Dr. Charles H. Parry, *in Reply to* Dr. Wilson Philip.

*To the Editor of the London Medical and Physical Journal.*

SIR,—I have this day, for the first time, seen a letter addressed to you, and inserted in your Journal so long ago as March 1820, in which Dr. Wilson Philip replies to Mr. Brodie, Dr. C. H. Parry, and others. My entire ignorance of the publication of such a reply, must be my apology for not having before noticed it. I am still ignorant what Mr. Brodie and others have done on this occasion; but, if the entire defence exhibits as weak an array of arguments as that portion of it which is devoted to my observations, they may, perhaps, have passed it over in silence. Thus far, indeed, my silence might also have been justified; but, as Dr. Wilson Philip imputes to me two errors, of which one is of an intellectual, and the other may be of a moral nature, I cannot feel satisfied without advancing the general plea—Not Guilty, as well to the charge of misapprehension as to that of misquotation.

To meet these accusations, it is necessary for me to analyse very minutely the whole of Dr. Wilson Philip's defence. Of the zeal and merits of this gentleman in the cause of science, every one is sufficiently convinced; but, if Dr. Wilson Philip is in error, and will not admit it, a close examination of the subject becomes necessary, exactly in proportion as his deserved celebrity may have a tendency to mislead others. There is no real charity to the world in the respect and deference which Dr. Wilson Philip appears to claim for science, under the circumstances in which he is placed.

Dr. Wilson Philip begins by saying, " It is with reluctance that I notice his answer, [Dr. C. H. P. in the Additional Experiments,] because I am wholly at a loss to account for his, I may almost say, uniform misconception of my meaning. In the 160th page he begins his answer, by observing, ' Dr. Wilson Philip, taking up the opinion of Bichât and others, has, indeed, attempted a direct proof from experiment in favour of the exclusive influence of the capillaries in carrying on the circulation.' If (continues Dr. Wilson Philip,) Dr. C. H. Parry can point out any passage in my treatise in which such an opinion is expressed, I shall be more surprised than he can possibly be that any one should maintain a position so extravagant."

This is, perhaps, one of the least successful attempts at the right conception of an author's meaning that ever appeared in support of the charge of almost uniform misconception. It shows to what extent, by a skilful separation from the whole context, an independent sentence may be made to convey any meaning which is suitable to a commentator's purpose. Will it be believed, that Dr. Wilson Philip endeavours to convey to your readers the idea that the author of the Additional Experiments charges him, Bichât, and others, with supposing the rest of the sanguiferous system wholly inactive towards the purposes of circulation? The veriest tyro would scarcely believe that such an assertion could have been made; and yet, if this is not Dr. Wilson Philip's belief, the intended and involved object of this quotation, his remark has no meaning at all. The alternative will, of course, not be admitted by those who know what are the opinions of Bichât, Dr. Wilson Philip, and others, in regard to the capillary circulation.

Dr. Wilson Philip is, then, honestly and fairly of opinion that, by " the exclusive influence of the capillaries" in all its connexions, is meant that this set of vessels performs the whole function of circulation through the system. So much for the first instance of the justice of Dr. Wilson Philip's remarks on the misconceptions of others.

Dr. Wilson Philip proceeds in his defence with a number of interrogatories, and asks, with an imposing air, " When have I maintained that the larger arteries do not possess an equal and proportional share of power with the capillaries?" I answer, every where; and venture to ask, in return, in what single instance he allows them to possess such a share of power?

At page 279 he says, " Dr. Parry maintains, in the 198th paragraph, that the blood is moved in the capillary vessels, not by the power of these vessels, but by the impulse it receives from the heart. This opinion it is not difficult to submit to the test of direct experiment:" and afterwards, p. 280, " that the motion of the blood in the capillaries chiefly depends on their own powers, appears from the following facts, which I have ascertained by repeated experiments."

If this is not something like denying " an equal and proportional share" of power to the larger arteries, I certainly have a complete misconception of the term *chiefly*, and should be grateful for any more accurate version of this passage.

Again, p. 280, " I have laid before the reader many experiments of a different nature, the results of which appear to be wholly incompatible with the opinion of Dr. Parry, and which seem to me to prove, in the most unequivocal manner, that the motion of the blood in the capillaries neither depends on the impetus given to it by the heart, nor on the contractility of the larger vessels."

Have we not in this passage, again, something like a denial that the power of the larger arteries is equal and proportional to that of the capillaries?

At p. 284, Dr. Wilson Philip says, " Does it not seem a necessary inference that the blood is moved in the capillaries by the power of these vessels themselves?" Has not this " necessary inference," too, something exclusive of " an equal and proportional," nay, of any share of power in other vessels?

The remark, however, of Dr. Wilson Philip at p. 280, must not be omitted: " That something must be ascribed to it (the contractile power of the large arteries,) cannot, I think, be denied." This is, to be sure, a very kind, though apparently unwilling, admission on the part of Dr. Wilson Philip, from which some important and extensive conclusions might, probably, be drawn against himself. On the present occasion I have only to remark, that if the " something" is not meant by him as implying an amount very different from " an equal and proportional" share, I confess that I have again misconceived him.

In the same tone Dr. Wilson Philip makes the second inquiry. "Where have I made any inference from the irregular motion of the blood, observed under certain circumstances in the capillaries, respecting the cause of circulation?" Respecting the cause of circulation! Is there not, in this passage, a little equivoque, analogous, in some measure, to that noticed at the beginning of this reply? Does Dr. Wilson Philip charge me with ascribing to him an opinion that the capillaries are the *cause of circulation?* and that these vessels have the power to circulate the blood through the system without the assistance of the heart and arteries? The cause of circulation! If such is Dr. Wilson Philip's honest belief, he is the last person who should accuse others of misapprehension. If he has any other belief, there is no occasion for any reply from me. There would be no difficulty in quoting Dr. Wilson Philip against Dr. Wilson Philip, through many pages. The alternative would in this, as in the former case, be perfectly unavailing.

Where, he asks, have I made any inference?—I answer, wherever the subject is discussed.

"As the motion of the blood in the smaller vessels begins to fail, where there is no inflammation, either in the living or in the dead animal, it is observed to stop and go on, to move backwards and forwards in the same vessel, and to stop in some vessels of the same part, sooner than others; phenomena which, it is evident, could not arise from the contractile power of the larger arteries."

Here, then, is no sort of inference? A careless reader might imagine that there was an implied, if not expressed, inference to this effect: the capillaries have an independant power by which they may act on the contained blood, and perhaps transmit it, when conveyed to them by the larger vessels. A hasty person might imagine something of this kind. We have the assurance of Dr. Wilson Philip that this is a complete misapprehension.

From what facts does this imaginary inference seem deduced? —from certain circumstances of irregular motion of the blood in the capillaries. This, too, we are informed, is a misapprehension.

"When the motion in the smaller vessels begins to fail!" "it is observed to stop and go on, to move backwards and forwards in the same vessel, &c." This, then, is quite a natural condition! there is in this case no irregularity of motion! it is, in short, quite a natural and regular course of things! Death is certainly a very natural and regular event; but inconsiderate persons had to learn that the circulation, after death, was devoid of irregularity; and that, in the animal distressed by experiments, the phenomena described were those of the regular circulation. Well, let it be so, if Dr. Wilson Philip is satisfied.

Dr. Wilson Philip proceeds with interrogatory the third—"Where have I maintained that no influence can be exerted through the heart and large vessels, by stimuli on the capillaries?" By stimuli on the capillaries! Where, Dr. Wilson Philip, have I said you maintained any such position? An author who vaguely charges others with misquotation, should not himself so seriously misquote.

The original passage in the Additional Experiments is this, —"In this account of his experiments, Dr. Wilson Philip's reasoning is of this kind: because the circulation in the capillaries is continued a certain time, (and I may add, in a certain way,) after the heart is removed, therefore, when the heart and large vessels remain, no influence can be exerted through them by stimuli; though these very stimuli, or sedative powers, are known to affect the heart and larger vessels."

Where is any thing resembling Dr. Wilson Philip's quotation of "by stimuli on the capillaries?" If he would take the trouble to use the consideration he so kindly recommends to me, he would find in this passage no such allusion, and might, perhaps, discover its meaning to be, that no influence on the capillaries can be exerted through the heart and large arteries by stimuli applied to, and known to affect, the heart and large arteries.

I enter no further into this subject in this place, because Dr. Wilson Philip does not deny the accuracy of my remarks as to the real tendency of his argument. Whenever an opportunity may occur, I shall be most willing to go into the particular question, and entertain any more philosophical and legitimate subject for discussion.

At page 165 in the Additional Experiments, I have quoted this sentence from Dr. Wilson Philip: "Can all the contractility of the larger arteries (the greater part of which, according to Dr. Parry's experiments, consists in the mere property of elasticity,) be destroyed by crushing the brain or spinal marrow?" To which I there reply, "The whole of this objection has its foundation in a careless examination of Dr. Parry's opinions, &c." Dr. Wilson Philip answers, "With respect to the quotation in page 165, if Dr. C. H. Parry will take the trouble to re-peruse it, he will find that I speak of the results of Dr. Parry's experiments, not of any thing which Dr. Parry has himself asserted." True; and on this very account I consider the inference a careless one. When the assumed result of these experiments depended partly on certain theoretical considerations, it would have been but candid and fair to admit the qualifications which, from the frequent repetition of his experiments, Dr. Parry had thought himself bound to adopt, or else to have given some reason for a contrary procedure.

Dr. Wilson Philip continues,—"Similar observations apply to other passages." These shadows he will not expect me to combat.

In his "Elements of Pathology and Therapeutics," p. 84, Dr. Parry had said, "Neither will this conclusion [that there is excessive momentum of blood in every palpable case of inflammation,] be invalidated, were it even proved, according to the opinion of Dr. Wilson, that the velocity of the blood in the vessels of an inflamed part is diminished, unless it be also proved that the velocity is diminished in a greater proportion than the quantity is increased."

Now, upon what does Dr. Parry insist in this passage?—that his conclusion as to excessive momentum is safe, even if the velocity of the blood in the vessels of an inflamed part be diminished, unless the velocity is diminished in a greater proportion than the quantity is increased: unless the former balance between these amounts be destroyed. Or, in other words, he admits that there would be no excessive momentum, if the quantity of blood were increased in a less proportion than the velocity is diminished.

Of the accuracy of this proposition I have elsewhere spoken. In this place I have only to remark, as in the Additional Experiments, that the whole admission is as to the rate of *momentum*, which would be altered, unless a lessened velocity were compensated by a proportional increase of quantity. It is clear that in all this there is not an iota of admission as to the fact of diminished velocity. But what does Dr. Wilson Philip make of it? "The truth," he observes, "of this inference appears, indeed, from direct experiments; for, from those made with a view to ascertain the state of the vessels in an inflamed part of which an account has been given, it clearly appears not only that the velocity of every part of the blood was lessened, (which Dr. Parry admits may be the case, supposing the lessened momentum arising from this cause more than compensated by the increased quantity of blood,) but that the general momentum of the blood also in the inflamed part was lessened," &c.

This sentence, on further consideration, is indeed much more curious than it appeared at first sight. Dr. Parry is made to admit that the *velocity* of every part of the blood may be lessened, supposing the resulting lessened momentum *more* than compensated by the increased quantity of blood,—that is, supposing the momentum to exceed its former amount. Thus, increase the quantity from 6 to 8, and reduce the velocity from 6 to 5, making the increase of momentum from 36 to 40, and Dr. Parry is made to suppose the velocity of every part of the blood may be lessened by an increased momentum arising from increased quantity! Is this believed to be perfectly intelligible to most men?

I proceed to the charge of misquotation brought against myself. The original sentence in the Additional Experiments is this:—"In the second place, when we consider that the momentum is the product of the quantity and velocity, it is not possible to suppose a lessened momentum more than compensated by an increased quantity of blood."

Dr. Wilson Philip answers, "The passage here given as a quotation from my treatise is, indeed, nonsense; but it is Dr. C. H. Parry's, not mine. The reader will observe, from the quotation in the last paragraph but one, that the passage in my treatise stands thus: 'Supposing the lessened momentum arising from this cause,'—namely, the lessened velocity,—more than compensated by the increased quantity of blood."

First, as to the misquotation. Though Dr. W. Philip has given his own sentence at large from the Additional Experiments, he has not the candour to call the reader's attention to the circumstance that, in the very same page, I had thus quoted him with perfect accuracy and distinctness, so that the supposed subsequent misquotation could, by no chance, have any improper influence on the judgment; and that the words he cites were only given as an abstract, easily filled up by the reader from the preceding correct quotation, but which were thus fairly employed, and quite sufficient to exhibit the real inaccuracy of the original sentence.

Dr. Wilson Philip admits that the passage here given as a quotation from his treatise is nonsense. I now repeat that the sentence, as it originally stands, and with all his additions, is incorrect; and ask, in what sense momentum can be compensated by quantity? He talks of the lessened momentum arising from lessened velocity being more than compensated by the increased quantity. Though the meaning may be guessed, the antithesis, under every qualification of it, is imperfect; and such want of precision should not have been admitted in a philosophical and argumentative treatise.

Dr. Wilson Philip again charges me with misquoting him at p. 174. There is certainly a typographical omission in this place. For "momentum of the whole part," read "momentum of the whole blood in the part."

But, does Dr. Wilson Philip earnestly and seriously believe that these three words were intentionally omitted? or does he feel seriously persuaded that my observations on this passage are the less correct, because these words should, and may be, inserted? Adopt them, and are the remarks then too severe? Would there have been any thing derogatory from the dignity of philosophy or the candour of science, had Dr. Wilson Philip first inserted the words accidentally omitted, and then considered whether the remarks applied or not? If they equally applied under both readings, would it have been too much to consider as an error in the press, that which, as an intentional omission, could answer no discoverable purpose? The mode of replication has very much the appearance of trifling.

On the subject of some of his experiments, Dr. Wilson Philip asks, "Is it fair, in repeating physiological experiments, to deviate from the method of the original experimenter in the most essential respects, and then adduce the necessary difference of result as a proof of his inaccuracy?"

Certainly nothing can be more unfair; but, after repeated failures in the employment of the prescribed method, it may be fair to attempt other means of coming at the predicted results. If, under the use of both means, the result be still different, it may not be unsafe to suspect some error in the original experimenter. This has been my course, and the suspicions excited must, at present, remain in their full force.

To Dr. Wilson Philip, for his kind advice, I return my hearty thanks. The example he proposes to me is such as I shall always be most proud and willing to follow. If, in the defence of this honoured person, I have been a little warm, my friends will excuse me; and the indifferent reader will, I trust, by this time, admit that the "fallacious reasoning" with which that acute author has been charged, must be opposed by arguments of a much more substantial nature than those which have as yet been adduced by Dr. Wilson Philip.

*Bath; May* 9, 1821.

